# A *de* novo duplication of chromosome 9q34.13-qter in a fetus with Tetralogy of Fallot Syndrome

**DOI:** 10.1186/s13039-016-0267-3

**Published:** 2016-07-25

**Authors:** Jing Liu, Hao Hu, Na Ma, Zhengjun Jia, Yuchun Zhou, Jiancheng Hu, Hua Wang

**Affiliations:** Prenatal Diagnosis Center of Province Hunan, The Maternal and Child Health Care Hospital of Hunan province, Changsha, Hunan 410008 People’s Republic of China

**Keywords:** TOF, 9qter duplication, SNP array, Non-invasive prenatal testing, Prenatal diagnosis

## Abstract

**Background:**

Partial duplications of the distal 9q have been rarely reported in literatures. The key features included characteristic facial appearance, long fingers and toes, slight psychomotor retardation, heart murmur et al. But rare severe congenital heart defects (CHD) such as TOF were reported to be associated with 9qter duplications.

**Case presentation:**

A 23-year-old woman was referred for genetic counseling and prenatal diagnosis at 25^3/7^ weeks of gestation due to her male fetus, diagnosed as Tetralogy of Fallot Syndrome (TOF) by prenatal ultrasound. SNP (Single nucleotide polymorphism) array revealed that the male fetus had a *de* novo 5.47 Mb duplication at 9q34.13-qter. Meanwhile, non-invasive prenatal testing (NIPT) using low coverage whole genome massively parallel sequencing of circulating cell-free fetal DNA (cffDNA) showed consistent results. Multiplex ligation-dependent probe amplification (MLPA) also confirmed the duplication at 9qter.

**Conclusion:**

In this paper, we present an Asian fetus with TOF caused by a *de* novo 5.47 Mb duplication at 9q34.13-qter. Duplication of 9q34.13-qter should be considered as an etiological diagnosis in the case of TOF. Our prenatal diagnostic findings provide important information for genetic counseling on the male fetus and future pregnancies in this family. Chromosomal microarray analysis (CMA) remains the first-tier clinical diagnostic test for prenatal fetus with suspicious syndromes. We also highlight the high potential application of NIPT in the screening of sub-chromosomal rearrangement.

## Background

Partial duplications of the long arm of chromosome 9 have been rarely reported in literatures. Turleau et al. first reported this new syndrome in 1975 [1]. The duplications of the distal 9q are even rare, since Alldedice et al first demonstrated the relationship between 9q34 duplications and phenotypic abnormalities [2]. The key features included slight psychomotor retardation, characteristic facial appearance, fingers and toes, heart murmur et al. But rare severe congenital heart defects (CHD) such as TOF were reported to be associated with these duplications. Amarillo et al. first reported the disrupted *RXRA* and complex microduplication of 9q (9q+) in a patient with TOF, suggesting that *RXRA* at 9q34.2 may be a critical locus in 9q + -associated CHD [3]. Here we present a male fetus with TOF who was found to have a *de* novo 5.47 Mb duplication at 9q34.13-qter detected by SNP array, which was confirmed by NIPT based on low coverage whole genome massively parallel sequencing and MLPA. A literature review was performed to refine correlation of CHD and 9q34 duplications. Our prenatal diagnostic findings provide important information for genetic counseling on the male fetus and future pregnancies in this family, and highlight the predictive values of NIPT in the screening of sub-chromosomal rearrangement.

## Case presentation

**Fig. 1:**
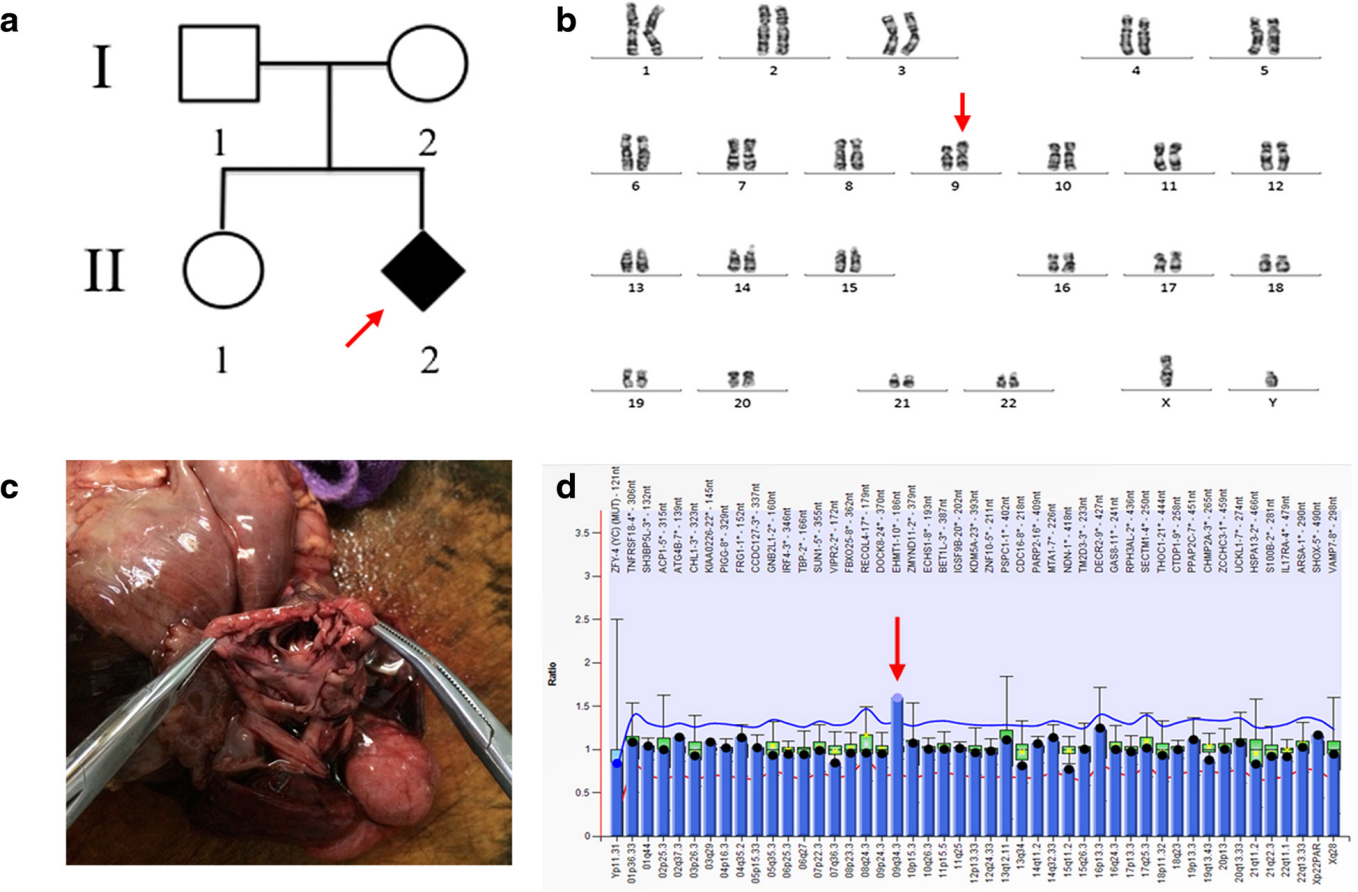
**a** The pedigree of this family. The red arrow indicates the proband; **b** G-banded karyotype of the affected fetus shows a inv(9)(p11q13) which was inherited from his mother; **c** Anatomical features of heart: VSD; **d** MLPA result reveals that the peak height ratio of patient at 9q34.3 is 1.5, which means a duplication at this region

A gravida 2, para 1 woman came to our clinical genetics center for genetic counseling at 25^3/7^ weeks of gestation. This is her second pregnancy. A first round prenatal ultrasound revealed that the male fetus (II:2) (Fig. 1a) had multiple defects: ventricular septal defect (VSD) and pulmonary stenosis. Biparietal diameter was 6.76 cm (50 ~ 95^th^ centile) and abdominal circumference (AC) was 23.0 cm (~95^th^ centile). A second round prenatal ultrasound was performed at 31 weeks of gestation, which indicated TOF.

The mother (I:2) was 23 years old and her husband (I:1) was 27 years old. Both of them were healthy non-consanguineous Chinese. Their first child (II:1) was a 2.5 years old girl, born at term by vaginal delivery after an uneventful pregnancy. Her birth weight was 3.4 kg (50 ~ 97^th^ centile). No significant history of anoxia was recorded at birth. When she was 1 year old, her parents found that her pupils of each eye were not in the same size. After a series of examinations, she was diagnosed as optic nerve hypoplasia, but no genetics testing was followed up.

Amino fluid and blood samples were obtained from the fetus and the family separately after receiving informed consent. According to the genetics diagnosis and ultrasound results, this pregnancy was subsequently terminated at 32 weeks, followed by an autopsy. The male fetus’ weight was 2052 g, height was 44.5 cm, head circumference was 30.6 cm, bust circumference was 26 cm, and AC was 25 cm(~5^th^ centile). Facial abnormalities:a round face with small jaw, normal eye distance (19 mm), flat nasal bridge, high-arched palate. Anatomical features of heart: VSD (Fig. 1c) and displacement of the aorta to the right side over the right ventricle (about 1/2). The right outflow tract (pulmonary) was 0.7 cm, the left outflow tract (aorta) was 1.2 cm, the thickness of right ventricle was 0.5 cm, left was 1.03 cm, bicuspid valve was 1.8 cm and tricuspid valve was 2.1 cm.

## Materials and methods

After culturing amino fluid cells and peripheral blood lymphocytes obtained from the fetus and the other three family members separately, routine chromosome G-banded (320–400 bands) karyotyping analyses were performed on metaphase cells according to standard protocols.

SNP array analysis: Genomic DNA was extracted from the amino fluid cells and peripheral blood lymphocytes by using DNA Extraction Kit (Tissue and cells) and QIAamp DNA Blood Mini Kit (QIAGEN, Hilden, Germany) separately. SNP array was performed using Affymetrix CytoScan®750 K Array (Affymetrix Inc, CA, USA), according to the manufacturer’s protocol. Array results were analyzed using Chromosome Analysis Suite (ChAS; version 2.1). All genomic coordinates were taken from the February 2009 (hg19) human reference sequence (NCBI Build 37). Genes and Online Mendelian Inheritance in Man (OMIM) references were from RefSeq and OMIM entries, respectively.

The maternal peripheral blood was collected into Cell-Free DNA BCT (Streck, Inc., Omaha, NE, USA) prior to amniocentesis and centrifuged according to the protocol. The plasma was shipped to Berry Genomics in Beijing for DNA extraction and downstream low coverage whole genome massively parallel sequencing for detection of fetal sub-chromosomal anomalies.

The SALSA MLPA P070 kit was performed to determine the DNA copy number of a single DNA sequence in each of 41 human subtelomeric regions. MLPA analysis was performed following the manufacturer’s instructions. The products were examined by ABI 3500dx genetic analyzer (Thermo Fisher, USA). Quantitative data were analyzed using the software of Coffalyser V8.0 (https://www.mlpa.com).

## Results

**Fig. 2:**
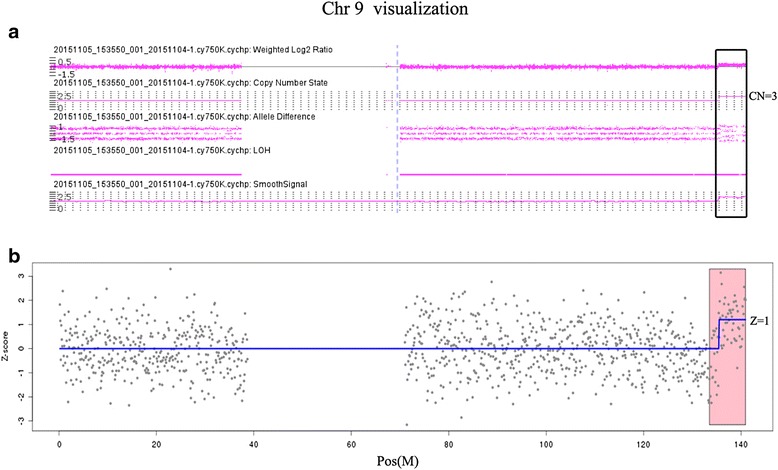
The ideograph of chromosome 9 in our patient with 9q34.13-34.3 gain. **a** CMA (Affymetrix ChAS Browser) detects a copy number gain at 9qter. The black frame region shows: 9q34.13q34.3(135,550,093-141,018,648) x3; weighted log_2_ ratio (WLR) is 0.5; copy number state (CN) is 3; allele differences (AD) (1.5, 0.5, −0.5, −1.5); smooth signal (SS) is 3; **b** NIPT via low coverage whole genome massively parallel sequencing of cffDNA in maternal plasma. Z score of the red bar region is 1, which means a copy number gain of this region

G-banded analysis of this family showed that the fetus inherited an inverted chromosome from his mother: 46, XY, inv(9)(p11q13), mat (Fig. 1b). The other two showed normal karyotypes. The SNP array result of the fetus was: arr[hg19] 9q34.13q34.3(135,550,093–141,018,648) × 3, 16p11.2(32,564,735–33,814,547) × 1 (Fig. 2a). The proximal duplication boundary was mapped within a 2.8-kb region between (nt. 135547269-135550093) at 9q34.13. The elder sister had a similar deletion at 16p11.2 as the fetus. No other pathogenic CNVs or variants of uncertain significance were identified in the parents. NIPT found a 5.6 Mb duplication in the fetus at 9q34 (Fig. 2b). MLPA analysis confirmed the duplication at 9q34.3 (Fig. 1d).

## Discussion

TOF occurs in three of every 10,000 live births. It is the most common cause of cyanotic cardiac disease in patients beyond the neonatal age, and accounts for up to one-tenth of all congenital cardiac lesions [4]. According to previous study, about 10 % of CHD in children with multiple congenital defects and/or intellectual disability are caused by trisomies 21, 18 and 13 [5]. However, none of these aberrations can be attributed to a single CHD phenotype and they are associated with different CHD at variable frequencies. With the development of CMA, aberrations smaller than 5 Mb can be detected and much more submicroscopic copy number variations were found to be associated with TOF, such as 22q11.2 deletion/duplication, 1q21.1 duplication, 1q32.2 deletion, 13q13.1–13.2 deletion et al. [6–8]. Genes disrupted by and within these CNVs thus represent excellent candidate genes for CHD/TOF.

In this case, the male fetus with TOF was found to have a *de* novo 5.47 Mb duplication at distal 9q34 and a 1.25 Mb deletion at 16p11.2. There was no OMIM gene within the deletion of 16p11.2. According to the database of genomic variants (DGV; http://dgv.tcag.ca), this deletion was thought to be a benign variant. 9qter duplications have been reported to be pathological for many times since 1975 [1, 2]. Until now, there were almost 50 cases reported in DECIPHER (Database of Chromosomal Imbalance and Phenotype in Human using Ensembl Resources), which overlapped with the 9qter region we have found. A 3.0–3.4 Mb region (9q34.11-34.13) was proposed to be critical for the presentation of several phenotypes associated with 9q34 duplications [9, 10]. The key features included characteristic facial appearance, long fingers and toes, slight psychomotor retardation, heart murmur et al.

We have compared the previously reported 9q34 duplications with similar sizes or within the region we detected. Besides other symptoms, congenital heart defects were not common among the 9q34 duplications (less than 1/3 of all the previous patients). Including our case, there were only three patients with 9qter duplications showing TOF. The other two reports were described in DECIPHER #284022 and by Amarillo [4]. Amarillo also conducted a literature review to refine the correlation of CHD and partial trisomy 9q, suggesting 9q34.2 region to be the critical locus for 9q + -associated CHD and *RXRA* to be a possible candidate gene for CHD. Another patient (DECIPHER #301594) with duplication of 9q34.13-34.2 (inherited from his unaffected father) loci that involves disruption of *RXRA* showed abnormality of the ventricular septum. This also provided an evidence to support *RXRA* to be a possible candidate gene for CHD. In our case, the 5.47 Mb interval contains 92 OMIM genes, including *RXRA*. However, the proximal breakpoint maps to the intron 1 of *GTF3C4* (*604892) at 9q34.13, which was first reported by Hsieh [11]. Until now, there is no evidence indicating its relationship with heart development.

According to previous reports, even the same duplications of 9q34 in the same family have different phenotypes [9], so phenotypic variabilities among patients with cardiac structural defects may be attributed to incomplete penetrance or epigenetic events. Additional expression studies are needed to determine the role of these genes and the region in the development of CHD.

The existence of cffDNA in maternal circulation [12] and the recent advent of massive parallel sequencing technologies [13] have enabled NIPT. Peters et al. first showed proof of concept that a fetal with a 4.2-Mb deletion on chromosome 12 between bands 12p11.22 and 12p12.1 can be identified by means of noninvasive analysis of DNA in maternal plasma [14]. Liang et al. also proved the feasibility of low-coverage massive parallel CNV sequencing for the diagnosis of chromosome disease syndromes [15]. Helgeson et al. developed a novel algorithm to identify fetal microdeletion events in maternal plasma. They tested 175,393 samples, and 55 sub-chromosomal deletions were reported. Their results demonstrated high positive predictive values for noninvasive testing of rare sub-chromosomal deletions [16]. In this case, we also applied the NIPT to detect the fetal sub-chromosomal arrangements, and we got a positive result. This work also proved that NIPT might be expanded into the detection of sub-chromosomal copy number variations.

## Conclusion

In this study, duplication of 9q34.13-qter should be considered as the etiology for this TOF. The results provide valuable information for genetic counselors to achieve molecular diagnosis of prenatal anomalies and make more accurate predictions about the clinical outcomes. CMA remains the first-tier clinical diagnostic test for prenatal fetus with suspicious syndromes. We also highlight the high potential application of NIPT in the screening of sub-chromosomal rearrangement.

## Abbreviations

AC, abdominal circumference; cffDNA, cell-free fetal DNA; CHD, congenital heart defects; CNV, copy number variation; MLPA, multiplex ligation-dependent probe amplification; NIPT, non-invasive prenatal testing; OMIM, Online Mendelian Inheritance in Man; SNP, single nucleotide polymorphism; TOF, Trilogy of Fallot Syndrome; VSD, ventricular septal defect
